# Supplementary material for: Integration of nested cross-validation, automated hyperparameter optimization, high-performance computing to reduce and quantify the variance of test performance estimation of deep learning models
Source: Comput Methods Programs Biomed. Author manuscript; Available in PMC 2025 Dec 3. (PMC12674930; doi:10.1016/j.cmpb.2025.109063)
Supplement: MMC1 [file NIHMS2112201-supplement-MMC1.pdf]

## S1. Implementation Details and Hardware Configuration

NACHOS and DACHOS were implemented using Python 3.8 and TensorFlow 2.6.2. They use `dill` for saving and loading configuration checkpoints, `mpi4py` for parallelization, `fasteners` for process locking and unlocking, `NumPy` for working with arrays, `scikit-learn` for computing performance metrics, `SciPy` for statistical analysis of the results, and `termcolor` for color-coded standard output messages. NACHOS and DACHOS are capable of generating learning curves, confusion matrices, and Receiver Operating Characteristic (ROC) curves for result visualization. They can also generate class activation maps for instance-wide prediction interpretation or feature importance , using GradCAM, which requires `Matplotlib` and `seaborn`.

NACHOS and DACHOS were designed to operate on both a supercomputer with GPU nodes and a Beowulf cluster of GPU workstations connected via a local Ethernet network. The algorithms were tested on the Schooner supercomputer, utilizing GPU nodes equipped with NVIDIA A100 GPUs. Jobs on the supercomputer were managed through the SLURM system, with the GPU count per node specified. Additional experiments were performed on a Beowulf cluster comprising GPU workstations with NVIDIA RTX A6000 and NVIDIA RTX 4090 GPUs, running Ubuntu 20.04.6 LTS. In the Beowulf cluster, data were distributed across all workstations, and jobs were executed using a configuration file that specified the GPU count and the IP address of each workstation. The repository for this paper can be fount at `https://github.com/thepanlab/NACHOS`.

## S2. Tables

Table S1: Performance comparison across different GPU types and configurations.

| GPU type | Memory bandwidth | # tensor cores | # GPUs | Time (hrs) |
|---|---|---|---|---|
| RTXA6000 | 112.5 GB/s | 336 | 1 | 21.9 |
| RTXA6000 | 112.5 GB/s | 336 | 2 | 11.1 |
| RTXA6000 | 112.5 GB/s | 336 | 3 | 6.9 |
| RTXA6000 | 112.5 GB/s | 336 | 4 | 5.4 |
| RTX4090 | 1000.0 GB/s | 512 | 1 | 13.8 |
| RTX4090 | 1000.0 GB/s | 512 | 2 | 7.0 |
| RTX4090 | 1000.0 GB/s | 512 | 3 | 4.7 |
| RTX4090 | 1000.0 GB/s | 512 | 4 | 3.6 |
| RTXA6000 + RTX4090 | - | - | 8 | 2.2 |
| A100 | 2000.0 GB/s | 432 | 1 | 11.7 |
| A100 | 2000.0 GB/s | 432 | 2 | 5.8 |
| A100 | 2000.0 GB/s | 432 | 4 | 3.0 |
| A100 | 2000.0 GB/s | 432 | 8 | 1.6 |
